# Fishery Induces Sperm Depletion and Reduction in Male Reproductive Potential for Crab Species under Male-Biased Harvest Strategy

**DOI:** 10.1371/journal.pone.0115525

**Published:** 2015-03-13

**Authors:** Luis Miguel Pardo, Yenifer Rosas, Juan Pablo Fuentes, Marcela Paz Riveros, Oscar Roberto Chaparro

**Affiliations:** Instituto de Ciencias Marinas y Limnológicas, Facultad de Ciencias, Universidad Austral de Chile, Valdivia, Chile; Institut Maurice-Lamontagne, CANADA

## Abstract

Sperm depletion in males can occur when polygynous species are intensively exploited under a male-biased management strategy. In fisheries involving crabs species, the effects of this type of management on the reproductive potential is far from being understood. This study tests whether male-biased management of the principal Chilean crab fishery is able to affect the potential capacity of *Metacarcinus edwardsii* males to transfer sperm to females. Five localities in southern Chile, recording contrasting crab fishery landing, were selected to assess the potential of sperm depletion triggered by fishery. Seasonally, male crabs from each locality were obtained. Dry weight and histological condition of vasa deferentia and the Vaso-Somatic Index (VSI) were determined in order to use them as proxies for sperm depletion and male reproductive condition. A manipulative experiment was performed in the laboratory to estimate vasa deferentia weight and VSI from just-mated males in order to obtain a reference point for the potential effects of the fishery on sperm reserves. Sperm storage capacity is significantly affected by fisheries; during the mating season vasa deferentia from localities with low fishery intensity were heavier than those from high intensity fisheries, and these differences were even more evident in large males. Histological section showed that this disparity in vasa deferentia weight was explained principally by differences in the quantity of spermatophores rather than other seminal material. VSI was always higher in males from localities with low fishery intensity. Males from localities with high fishery intensity showed little capacity to recover sperm reserves and the VSI of these males remained below the values of the just-mated males. Detriment in the capacity of males to transfer sperm is the first step to sperm limitation in an exploited population, thus detection of sperm depletion can be an alert to introduce changes in the current management of crabs.

## Introduction

Fishery activity is a source of selective mortality for wild populations. This is more evident when management measures target a specific size and sex, changing the structure of the population under exploitation. This is the case in most crab fisheries, which typically select for large, dominant males. This selective pressure leads to changes in sex ratios in favor of females [1] and possibly sperm limitation [2–4].

Most crabs display a polygynous mating system; therefore one male may mate with more than one female within one reproductive season. When dominant males are scarce due to fishery pressure, remaining males mate repeatedly in order satisfy the sperm demand of females [5] and potential for sperm depletion appears.

The capacity of males to transfer sperm is limited by their sperm reserves and the rate of sperm regeneration [6]. In crab species where sperm depletion has been studied, after copulation male recovery of sperm reserves varies in time, from a few days to over one year, depending on water temperature, food availability and population density [6–9]. On the other hand, in several species groups including crabs, males are able to control the amount of sperm transferred to females by regulating the ejaculate load allocated; this control has been associated principally with the reproductive potential of females, number of mating opportunities [8] and the risk of sperm competition [10]. Variable sperm recovery times and sperm allocation strategies are strong evidence that sperm production is not unlimited or cheap [11]. In consequence, males from diverse taxa are prudent in their use of sperm reserves [10].

Detection of sperm depletion in a harvested population is relevant, as it can be the primary cause of sperm limitation and subsequently low reproductive success in females. However, sperm depletion is not easy to estimate in wild populations, because the recent mating history of males is unknown and due to difficulties in obtaining a base line to establish when sperm reserves have really been depleted.

Decapod crustacean males have paired testis and vasa deferentia leading to paired male sexual openings [12]. Sperm development occurs completely or partially in the testis, but the vas deferens is where mature sperm are packaged into spermatophores and embedded in seminal liquid [12]. Therefore, vasa deferentia are the sperm storage structures used before transfer to females. In fact, Sainte-Marie [6] proposes that relative weight of the vasa deferentia with respect to crab male body is an indicator of sperm transference capacity, which can be expressed as a Vaso-Somatic Index (VSI). This index accounts for variation in sperm reserves and is useful for inter and intra specific comparison. For example, variation in the VSI among males from stocks under fishery pressure could be evidence of the effect of fishing on sperm transfer capacity.

Laboratory experiments using controlled mating can contribute to reveal how much of sperm reserves (expressed as vasa deferentia weight or VSI) is transferred to the female during one or multiple matings and how much time is required for males to recover their sperm reserves. In this way, field monitoring at appropriate spatial and temporal scales in conjunction with laboratory experiments can help to assess the effect of male-biased fishery management on the reproductive condition of male crabs.

*Metacarcinus edwardsii* (BELL 1835) is the most important commercial crab for the Chilean artisanal fishery, with a mean annual landing around 5000 tons over the last five years (Chilean national fishery statistics, SERNAPESCA). Their exploitation is concentrated in the south of the country (40–48°S), but with large variation in local fishery intensity [13]. At present, for this region, crab fishery is managed with a minimal legal size (110 mm carapace width, CW) for both sexes and harvesting of females is banned while they are carrying embryos (around 4–5 months, usually from April to August). So, ovigerous females must be released when caught in traps. This is a year-round fishery with no seasonal closures.

Despite its economic importance, reproductive aspects of *M*. *edwardsii* are far from being well known. The information available focuses mainly on females [14,15]. This crab is a univoltine species with a mating season from October to January, which is associated with female molt. After mating, females can store sperm inside the seminal receptacle for several months to fertilize oocytes, then carry embryos during autumn and winter [15]. For males, current information is restricted to sexual maturity: 50% morphological maturity (where 50% of males reach maturity) has been recorded at 118 mm CW but successful mating can occur at a size of 101 mm CW, corresponding to 50% physiological (gonadal) maturity [14]. Sexual dimorphism is only evident in the shape and size of abdomen, and after morphological maturity, in the size of chelae [14]. The effect of selective fishing (sex and size) on the reproductive condition of *M*. *edwardsii* is unknown to date.

In this study, we test whether high harvest levels with a strong bias for large males are associated with sperm depletion in crab populations. In order to evaluate this supposition, we conducted experiments under natural and laboratory conditions. First, a seasonal monitoring of the VSI, weight and histological condition of vasa deferentia was performed in five localities (two with small crab landings and three with large crab landings). Then, in order to obtain a comparative base-line, under controlled conditions in the laboratory, the weight of the vasa deferentia and VSI after one mating was estimated from males with one ablated gonopod.

## Materials and Methods

### Field sampling

**Fig 1:**
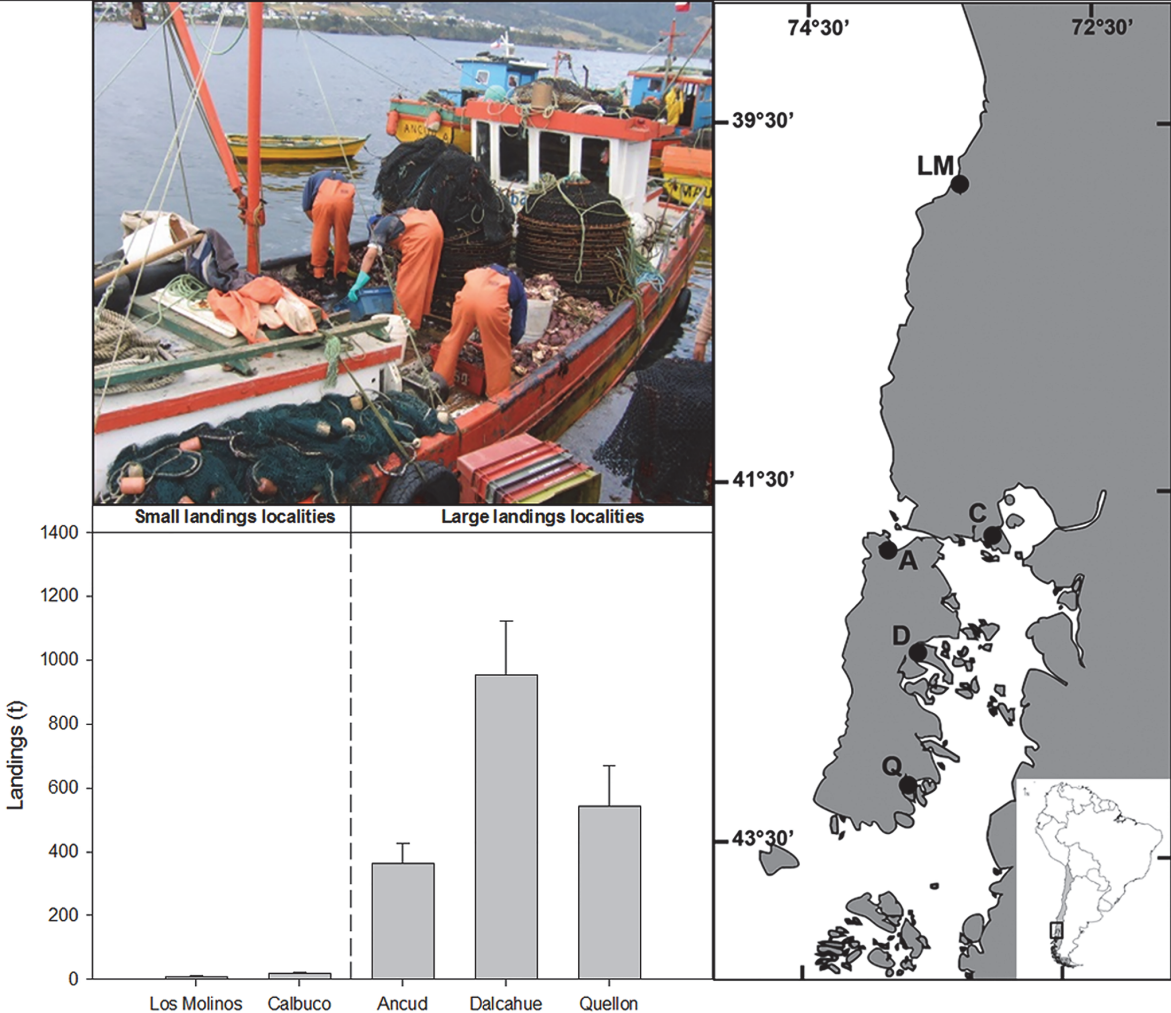
Left panel: Sampling localities at the southern of Chile; LM: Los Molinos, C: Calbuco, A: Ancud, D: Dalcahue, Q: Quellon. Right panel: Mean and standard error of *Metacarcinus edwardsii* landings over the last 5 years by locality, and picture showing a typical boat involved in the crab fishery and fishermen working during a landing.

Five localities in southern Chile (Fig. 1) were chosen based on the official statistics of target crab landings and categorized as having either small or large landings. We considered in the small landing category: Los Molinos (39°51′16.7″ S; 73°23′40.3″W) and Calbuco (41°45′47.1″ S; 73°05′20.1″W), where the mean landing had been less than 0.25 ton per year over the last five years. On the other hand, in the large landing category we included: Ancud (41°50′59.8″ S; 73°51′32.5″W), Dalcahue (42°22′46.3″ S; 73°35′42.5″W) and Quellón (43°08′18.4″ S; 73°36′43.4″W), locations with mean landing varying between 250 to 600 ton per year over the last five years (Fig. 1). Thus, recorded differences between small and large crab landings were up to two orders of magnitude. For this fishery, activities are concentrated around a landing port, where operative distances to fishing sites ranged from 18 km (Quellon) to 40 km (Dalcahue) from ports [16], therefore landing statistics are a good proxy of the local fishing pressure. Independent fishery monitoring of *M*. *edwardsii* corroborates the crab landing differences among localities [13].

During a field survey at each locality, between 13–30 physiologically mature males (>101 mm, CW, see [14]) were caught with commercial traps and transported to Laboratorio Costero Calfuco at the Universidad Austral de Chile. Four seasonal field trips were carried out repeating this sampling procedure, in fall (April 2012), winter (July 2012), spring (September 2012) and summer (February 2013). In the laboratory, specimens were measured (carapace width = CW) and dissected in order to carefully extract both vasa deferentia. Then all samples were oven dried for 24 h at 75°C and weighted to a precision of 0.0001 g. The crab bodies (without legs and chelae), were also oven dried for 5 days at 75°C and then weighted to a precision of 0.01 g. The Vaso-Somatic Index was calculated as suggested by Sainte-Marie [6] and expressed as a percentage: VSI = (VDW/BDW) * 100, where VDW is the mean dry weight of the two vasa deferentia and BDW is the dry weight of the body.

### Direct estimation of abundance, sex ratio and crab size by localities

In order to evaluate if small and large landings reflect differences in fishing intensity rather than differences in crab population abundance or size structure, a direct assessment by SCUBA diver was performed in all localities included in the study. Five transects (15 m) per season (total n = 20) and locality were used to estimate the abundance, size and sex ratio of crabs. All localities were visited each season, but transects were not located at the same points each visit. Transects were conducted approximately 10 m apart from each other. Abundance and mean crab size were estimated including all legal sized crabs on transects and separated by sex.

The sex ratio was estimated from the reproductively mature section of the population, represented by females and males larger than 101 mm CW [14]. This size corresponds to physiological maturity, where the 50% of individuals were able to produce gametes and can potentially participate in mating [14]. Therefore, only crabs collected during the mating season were used in the analyses. Since females are receptive to mating only for a few days after they molt and do not molt synchronously over a short time period, this sample does not represent an operational sex ratio *sensu stricto*, but is an approach to it.

### Fishery intensity index

Given that reliable estimates of exploitation rate and/or fishery effort are not available for all these localities, an index of fishery intensity was constructed. This index was calculated as the ratio of mean annual landings to legal crab abundance (>110 mm CW) per locality.

$$\text{Fishery intensity index}={\text{L}}_{\text{B}}/{\text{A}}_{\text{B}}$$

Where L_B_ = landing expressed in biomass (ton); A_B_ = local abundance expressed in biomass (ton). The numerical abundance of crabs obtained in transect samples was converted to crab biomass using the size-weight relationship estimate for *M*. *edwardsii* for Chiloe Island [17];

For females; W = 0.000511 * CW^2.762^ (r = 0.90; n = 3.425)

For males; W = 0.000074 * CW^3.17^ (r = 0.94; n = 4741)

Where W = wet weight; CW = carapace width

This index indicates the intensity of fishing activity in a specific locality with respect to local crab abundance and assuming that subtidal transect surveys were representative of average crab abundance over the fished area at each locality.

### Histological and monthly assessment of vasa deferentia condition

To examine the histological condition of vasa deferentia among localities with contrasting fishing scenarios, 10 males per locality per season were dissected, the right vasa deferentia was extracted and fixed in Bouin solution for at least two days. Then, samples were sequentially passed through a 50–70–80–96–100% ethanol series for 30 min each, 100% ethanol–buthylic alcohol (1: 1 v/v) for 30 min and buthylic alcohol for 25 min (twice). Samples were embedded in paraffin and serial sections of 6 μm were cut and stained with hematoxylin-eosin. The condition of vasa deferentia was determined by the estimation of area covered by spermatophores in the vasa deferentia lumen, expressed as a percentage. This estimation was done in the middle section of each vasa deferentia, using microscopic photos analyzed using CPCe 4.1 software.

Also we performed a monthly monitoring of VSI. Each month over one year (September 2011 to August 2012), thirty physiologically mature males were collected from Los Molinos, measured, dissected and VSI estimated immediately after capture.

### Laboratory manipulative experiment

Approximately one hundred physiologically mature males and one hundred females (larger than 75 mm CW in order to increase the probability to obtain receptive females) were collected during early spring 2012 from Los Molinos. The specimens were transported to the laboratory and separated by sex in 500 l tanks with flowing sea water, air supply and *ad libitum* food (mussels). The right gonopods were ablated (unilateral ablation) in most males and remaining individuals were bilaterally ablated. Ablation was performed with a clean cut at the base of appendages with surgical scissors. One week later, bilaterally ablated males were used as sentinels to detect receptive females (just before molt) in the female tank. Receptive females were detected daily by observing the guarding behavior of these males.

Once receptive females were detected (n = 54; mean CW = 100.8 ± 13, size range = 78.8–140 mm CW), they were paired with a unilaterally ablated male in a 100 l tank (n = 54; mean CW = 137.4 ± 12, size range = 112.9–164.6 mm CW). After copulation and the post guarding period, males were dissected and the weight of the right (full) and left (depleted) vasa deferentia were measured. Since male crabs store seminal fluid and sperm (packaged in spermatophores) in the vasa deferentia before copulation, in order to estimate the level of sperm depletion, the amount of seminal material delivered (i.e. ejaculate) during a mating event was calculated based on the difference in weight between right and left vasa deferentia from unilaterally ablated males just after mating. A similar technique has been used with the blue crab *Callinectes sapidus* [18]. This estimation assumes that both vasa deferentia have similar weight before mating. Fifty-nine just-caught males from Los Molinos were used as a control to test this assumption. VSI for just-mated males was estimated using only the weight of left vas deferens. Female size was not related to weight of ejaculate delivered (R^2^ = 0.03), which was determined by the difference in weight between the left and right vasa deferentia.

### Statistical analyses

First, linear regressions were performed to test the dependence of vasa deferentia weight and VSI on crab size. Then, in order to detect differences in vasa deferentia weight during the mating season (spring and summer), a one-way ANCOVA was performed, using provenance as factor (laboratory, high and low fishery) and carapace width as covariate. In the case of VSI, a one-way ANOVA with the Welch procedure [19,20] was performed to compare localities each season. This analysis was preferred to a two-way ANOVA given that no correction was found for variance heterogeneity in VSI data. For the monthly monitoring data, one-way ANOVA was used to compare the mean value of VSI for each month. To test differences in the area covered by spermatophores in the vasa deferentia (%) among localities and season a two-way ANOVA was performed. Also a t-test for dependent (paired) data was used to compare left and right vasa deferentia weight from experimentally ablated just-mated males, the same analysis was performed for the control group. For data obtained from subtidal transects, differences in crab abundance among localities were tested by a one-way ANOVA with the Welch procedure. To detect differences in size between sexes and among localities a two way ANOVA was performed. In both cases, a Duncan test for pair-wise comparison was used. The deviation from a 1:1 sex ratio was tested by locality using an observed versus expected chi-square test.

In all analyses, test assumptions were evaluated with the Cochran test for variance homogeneity and Shapiro-Wilks for normality. All analyses were run using R and STATISTICA software.

### Ethics Statement

Species involved in this research are not endangered or protected.

## Results

**Fig 2:**
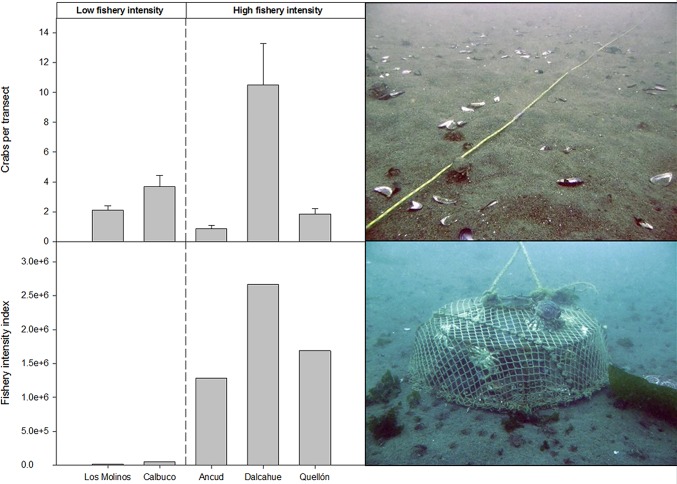
Upper panel: Abundance of legal sized crabs (>110 mm of carapace width) by locality, all seasons and transects pooled (n = 20), bars representing standard errors; picture showing a 15-m dive transect. Lower panel: Fishery intensity index by locality (see details in text) and picture showing the typical crab trap used to catch *M*. *edwardsii* in southern Chile.

Local crab abundances showed large differences (Dalcahue showed significant differences with all other localities; ANOVA, F_4,90_ = 9.4, p<0.001), which were not associated with crab landing category (Fig. 2). However, the five target localities presented large differences in fishery intensity (Fig. 2), matching landing categories; Ancud, Dalcahue and Quellon (large fishery landing) showed notably higher values of fishery intensity than Los Molinos and Calbuco (low fishery landings).

**Fig 3:**
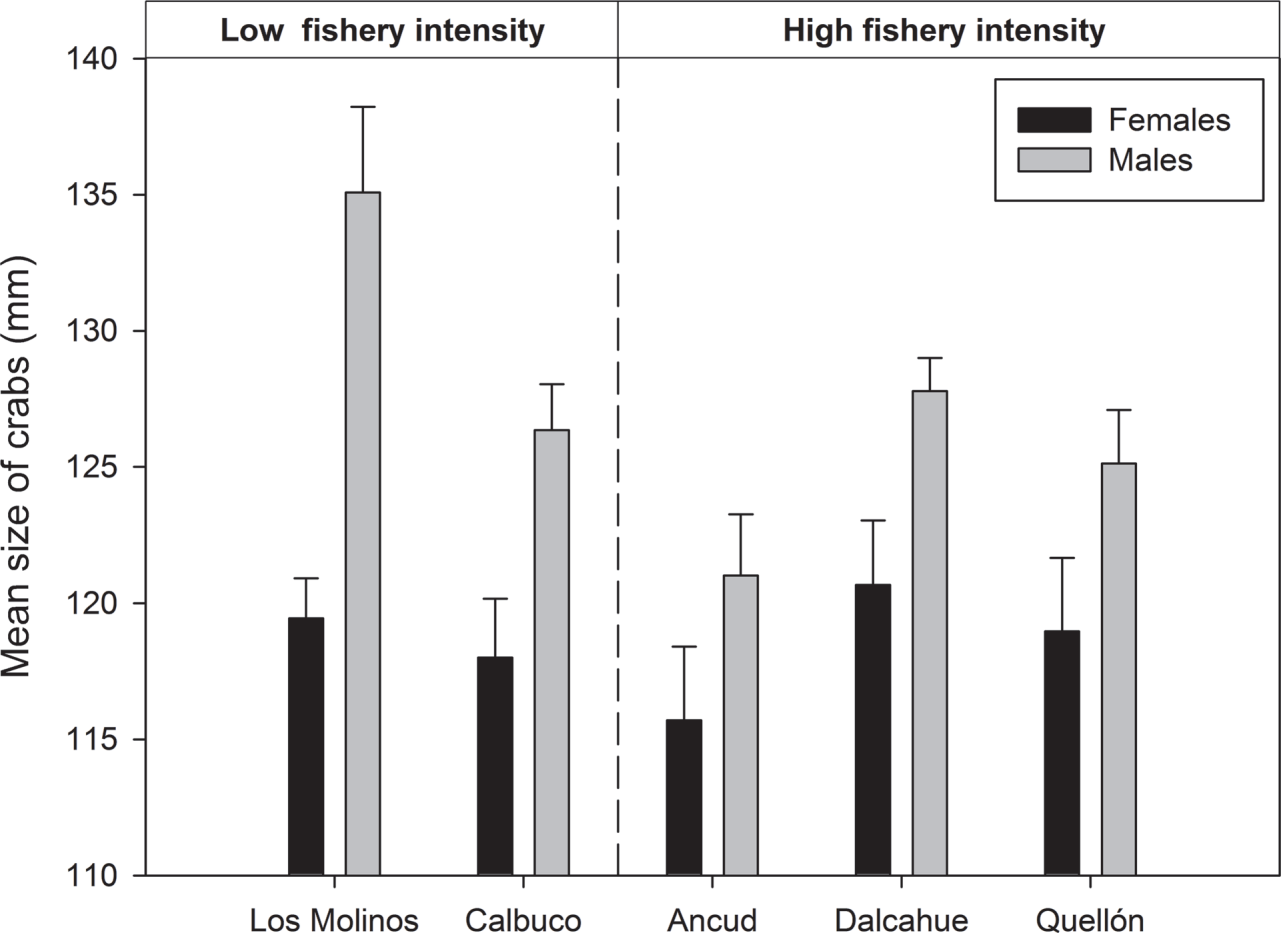
Mean carapace width by sex of legal sized crab (>110 mm) determined from subtidal dive transect (15 m) by locality. Los Molinos and Calbuco are categorized as low fishery intensity and Ancud, Dalcahue and Quellon are categorized as high fishery intensity. All seasons pooled. Bars represent standard errors.

With respect to crab size, males were larger than females in Calbuco, Los Molinos and Dalcahue (Duncan post hoc test; p<0.001), however at Ancud and Quellon both sexes had non-significant differences in size (Duncan post hoc test; p = 0.4 and 0.2 respectively). Males from Los Molinos were larger than in all other localities (Duncan post hoc test; p< 0.05), except Dalcahue (Duncan post hoc test; p = 0.1) (Fig. 3).

**Fig 4:**
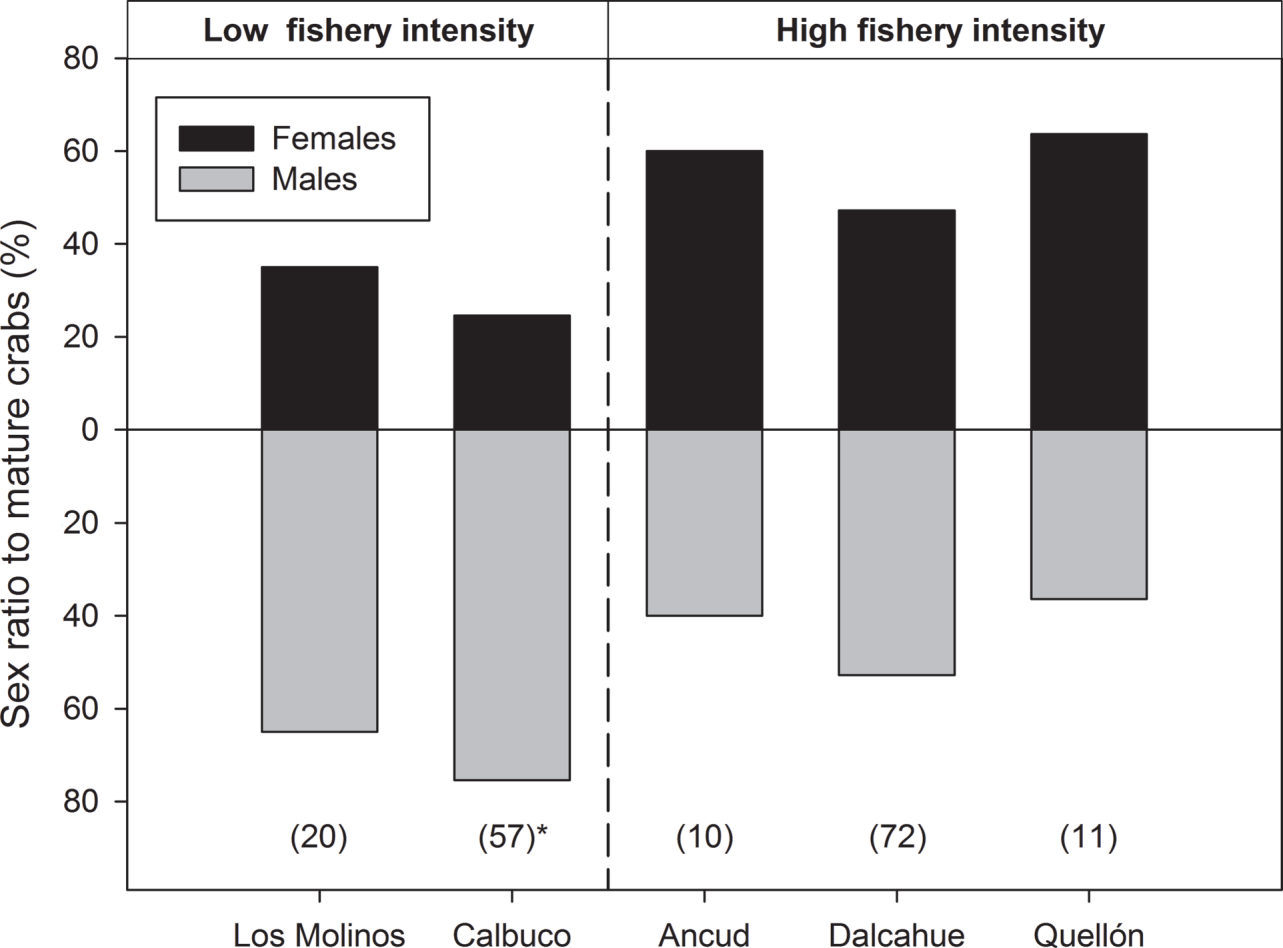
Percentage by sex of *Metacarcinus edwardsii* crabs counted in visual transects (15 m) by SCUBA divers. Crabs were sampled during mating season at five localities in Southern Chile, only physiologically mature crabs (>101 CW) were considered. Total number of crabs by locality in parenthesis, asterisks indicate significant deviation from the 1:1 sex ratio (p<0.01).

Deviation from a 1:1 (male:female) sex ratio for physiologically mature crabs was only significantly detected in Calbuco (3.1 males: 1 female, χ^2^ = 10.6, p = 0.03, n = 5), while the other locality with low fishery intensity, Los Molinos, had similar tendency (Fig. 4) but deviation was not significant (p>0.05). All localities in the high fishery intensity category (Ancud, Dalcahue and Quellon) recorded non-biased sex ratio for mature crabs (Fig. 4).

**Fig 5:**
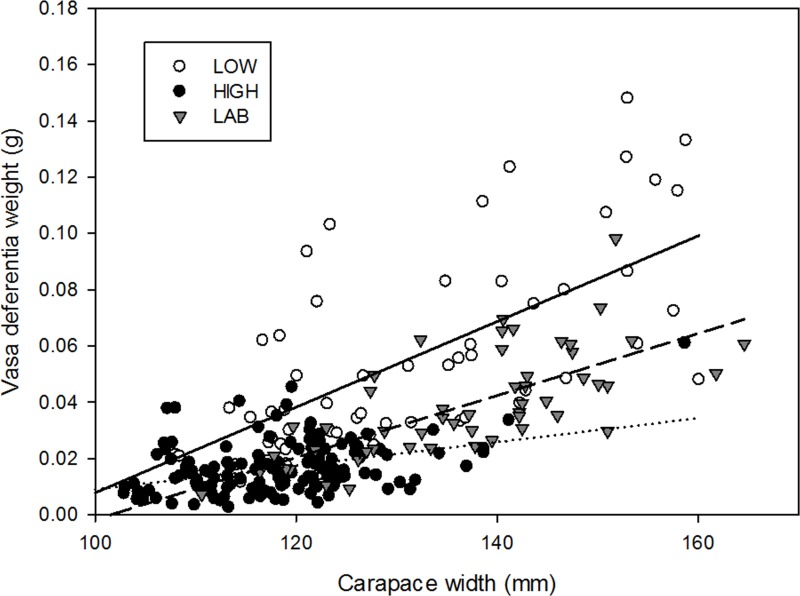
Relationship between carapace width and vasa deferentia weight of *Metacarcinus edwardsii* males during the mating season for individuals from contrasting fishery scenarios (low and high fishery intensity) and just-mated males from laboratory experiments. Data from localities with similar fishery intensity were pooled. Continuous line; linear fit from low fishery intensity. Dashed line; linear fit from laboratory experiment, dotted line; linear fit from high fishery intensity.

Weight of vasa deferentia during the mating season with different provenance (from localities with high—low intensity fishery or laboratory) showed differences in linear regression fit. Provenance and crab body size showed a significant interaction (ANCOVA, Provenance * CW: F_2,255_ = 20.8, p = <0.001), indicating that slopes were non-homogenous (Fig. 5). While males from low intensity fishery localities and laboratory experiments had similar slope values (regression, low intensity fisheries: F_1,63_ = 64.5, p<0.001, slope = 0.0015, r = 0.71; Just-mated males from laboratory: F_1,52_ = 47.1, p<0.001, slope = 0.0011, r = 0.69), males from the high intensity fisheries had a much smaller slope value and poor but significant fit to a linear model (regression, high intensity fisheries: F_1,144_ = 35.3, p<0.001, slope = 0.0004, r = 0.44).

As expected, left vasa deferentia weight was lighter than right for experimental unilaterally ablated males (one-tailed paired t-test, T_1,52_ = 9.63, p<0.001), but this was not the case for non-ablated control males (two-tailed paired t-test, T_1,58_ = 0.01, p = 0.92). Differences in vasa deferentia weight of ablated males indicated that in one mating event, males deliver a mean of 15% (range 2.8–49%) of the vasa deferentia weight to a female. This percentage is not related to male size within the size range used (Regression, F_1,52_ = 1.49, p = 0.23, r = 0.17) or to female size (Regression, F_1,52_ = 0.25, p = 0.34, r = 0.14).

**Fig 6:**
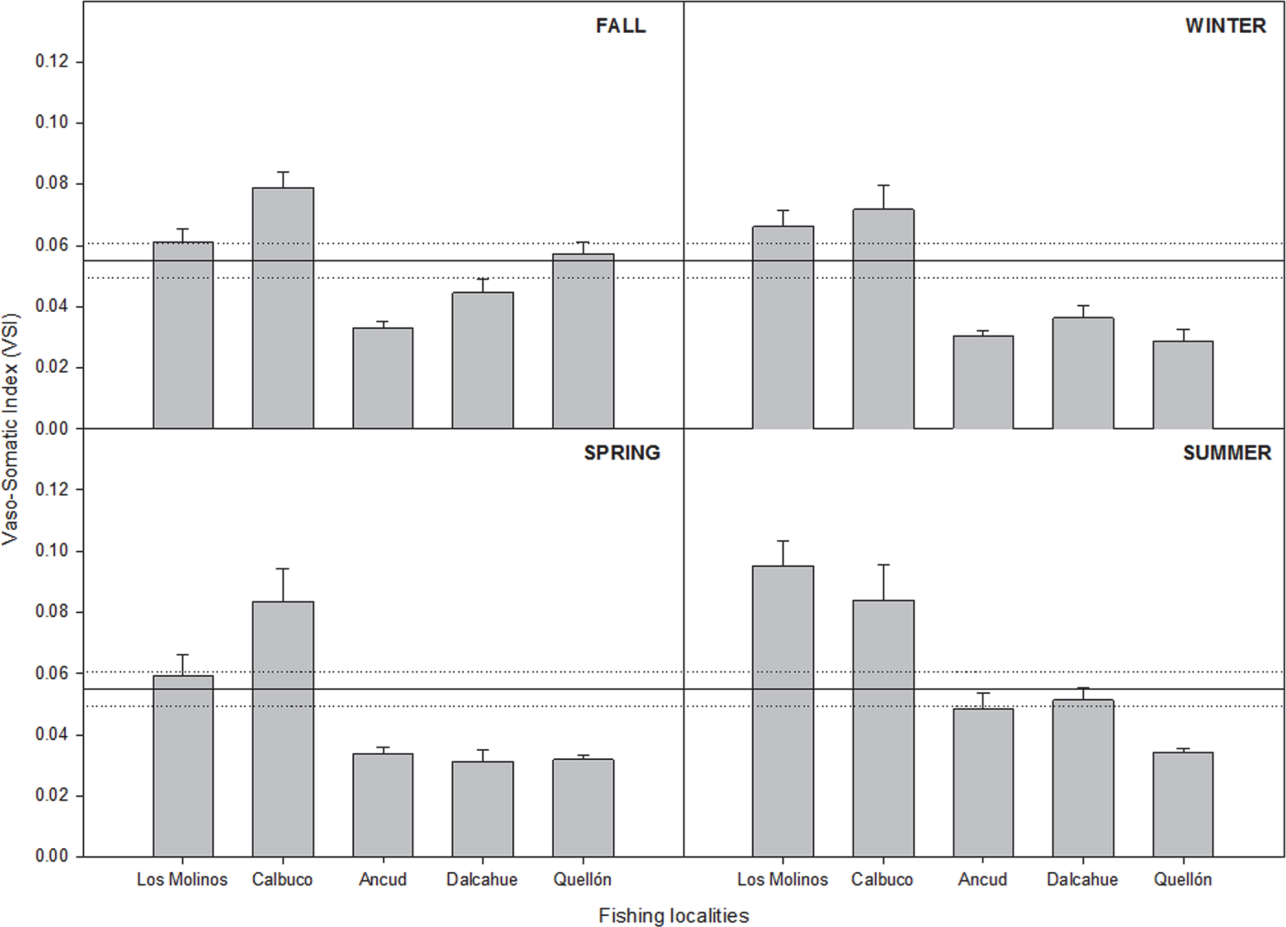
Seasonal mean Vaso-Somatic Index (VSI) and standard errors for *Metacarcinus edwardsii* males from localities with contrasting fishery intensity. Lines indicate mean (continuous) and confidence limit (95%, dashed) of VSI from just-mated laboratory males. Los Molinos and Calbuco are categorized as low fishery intensity and Ancud, Dalcahue and Quellon are categorized as high fishery intensity.

The VSI showed significant differences among localities, regardless of the season analyzed. In all cases, the two low fishery intensity localities had higher values of VSI in comparison to high fishery intensity localities (Fig. 6). Within localities of the same category differences were not found, except between Ancud and Quellon in fall and Los Molinos and Calbuco in spring (Table 1). In general, the VSI of males from high intensity fishing localities was 49% lower than those from low intensity fishing localities. The values were poorly dependent on male CW (Regression, F_1,551_ = 55.1, p = 0.001, r = 0.301). The VSI of males from experimental matings exhibited a range of 0.023 to 0.146, with a mean of 0.055 (Confidence Interval_95%_ = 0.049–0.061, n = 53) (Fig. 6).

**Table 1:** Analysis of variance and pair-wise comparison of Vaso Somatic Index from localities with contrasting fishery intensity by season.

One way ANOVA with Welch procedure					
SEASON	Catch date	n	df	F	p
FALL	April 24 to May 9	153	4	24.88	<0.001
WINTER	July 18 to August 3	143	4	15.6	<0.001
SPRING	November 1–4	130	4	9.05	<0.001
SUMMER	February 18–20	126	4	22.02	<0.001
**Pair-wise t comparison with Bonferroni adjustment**					
**FALL**	n	Molinos	Calbuco	Ancud	Dalcahue
Los Molinos	30	—			
Calbuco	30	0.022	—		
Ancud	31	<0.001	<0.001	—	
Dalcahue	30	0.05	<0.001	ns	—
Quellon	32	ns	<0.001	0.001	ns
**WINTER**					
Los Molinos	28	—			
Calbuco	28	ns	—		
Ancud	32	<0.001	<0.001	—	
Dalcahue	28	<0.001	<0.001	ns	—
Quellon	27	<0.001	<0.001	ns	ns
**SPRING**					
Los Molinos	17	—			
Calbuco	33	<0.001	—		
Ancud	31	<0.001	<0.001	—	
Dalcahue	21	<0.001	<0.001	ns	—
Quellon	28	<0.001	<0.001	ns	ns
**SUMMER**					
Los Molinos	23	—			
Calbuco	13	ns	—		
Ancud	30	<0.001	0.002	—	
Dalcahue	30	<0.001	0.006	ns	—
Quellon	30	<0.001	<0.001	ns	ns

Los Molinos and Calbuco categorized as low fishery intensity. Ancud, Damcahue and Quellón categorized as high fishery intensity.

**Fig 7:**
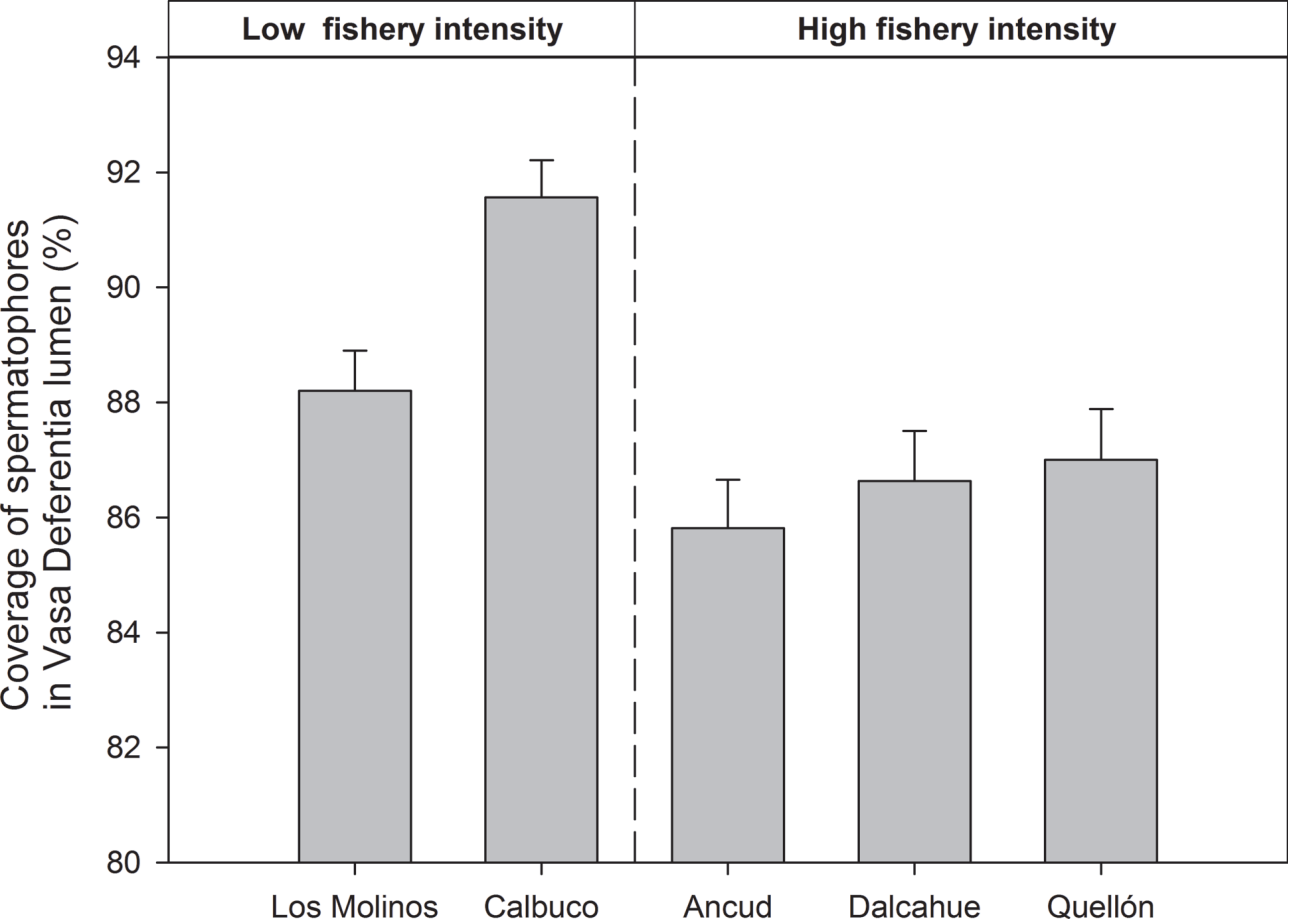
Mean area covered (%) and standard errors by spermatophores at the middle section of vasa deferentia from males of *Metacarcinus edwardsii* from localities with contrasting fishery intensity (high and low fishery localities). Data from 339 histological sections, seasons pooled.

The histological condition of vasa deferentia (% of coverage of spermatophores in the vasa deferentia) also showed differences between localities with low and high fishing intensity, but were less evident than VSI data (ANOVA, Localities: F_4,302_ = 9.71, p<0.001). The histological sections from Los Molinos and Calbuco showed a higher percentage of spermatophore coverage demonstrating a higher proportion of vasa deferentia with full sperm reserves (Fig. 7). At seasonal scale, spring showed the lowest spermatophore coverage in vasa deferentia, which coincides with the crab´s mating season (ANOVA, Season: F_3,319_ = 3.4, p = 0.018).

**Fig 8:**
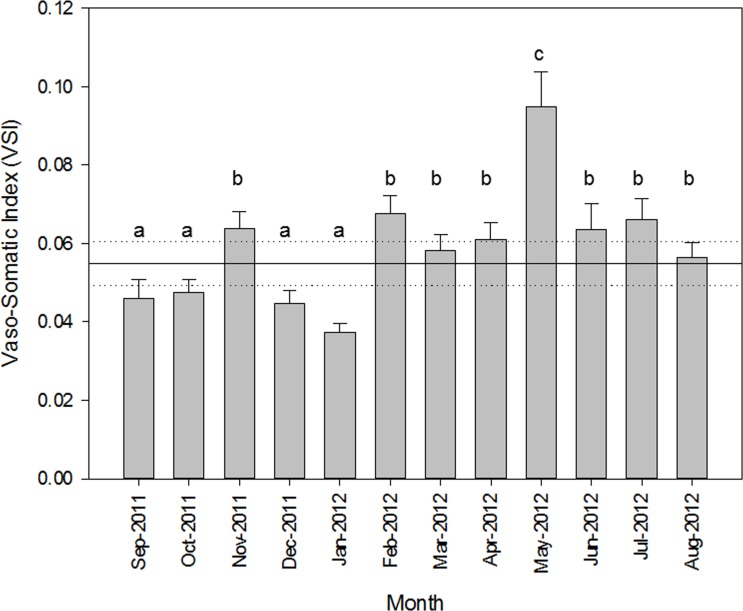
Monthly variation in Vaso Somatic Index (VSI) and standard errors from males of *Metacarcinus edwardsii* from Los Molinos. Lines indicate mean (continuous) and confidence limit (95%, dashed) of VSI from just-mated laboratory males. Letters indicate significant differences (p<0.01).

Monthly monitoring of VSI in Los Molinos showed significant seasonal variation (ANOVA, Month: F_11,302_ = 8.9, p<0.001) (Fig. 8). During spring months VSI was relatively constant, decreasing in summer (December and January) and recovering higher values in February.

## Discussion

Fishery activity has been shown to affect the sex ratio of mature crabs during the mating period, males tend to be more abundant than females in localities with low fishery intensity but this is not the case in localities with high fishery intensity. On the other hand, differences in mean size between male and females were absent in most the higher fishery localities. Also, large males were only found in one of the low fishing localities. Both effects could lead to sperm depletion by increasing the levels of polygyny.

Potential sperm transfer capacity of male crabs, inferred from observed VSI, was affected by the male-biased fishery. Large differences were detected among localities with contrasting fishery intensity. Vasa deferentia weight and the vaso-somatic index showed that the reproductive potential is diminished in males, and especially so in large males, from localities where intense fishery exploitation had been recorded.

Vasa deferentia increased in weight with male body size, as has normally been described in crabs [6]. However, the relationship between size (carapace width) and vasa deferentia weight varied in the magnitude of the slope and fit to linear models depending on male provenance. During the mating season, males from low fishing localities show a higher slope, and the vasa deferentia increase 2 mg per mm of carapace width, while males from high fishing localities showed only a 0.4 mg increase and a low fit to the expected positive linear relationship, because large males showed relatively little additional seminal material with respect to their size. Just-mated males (a single mating) had an intermediate trend with respect to high and low fishery localities.

The experimentally controlled mates can be taken as a baseline to evidence sperm depletion in crabs (as in [21]). In this sense, strong evidence for sperm depletion is observed in localities with higher fishery intensity. Dry weight of seminal material is determined principally by sperm (aggregated in spermatophores) and seminal liquid, but histological analysis indicate that males from high fishing localities had lower sperm reserves in their vasa deferentia (i.e. a lower area covered by spermatophores with respect to vasa deferentia lumen), supporting the idea of sperm depletion rather than differences in seminal liquid.

The amount of sperm transferred to females depends on several environmental conditions and individual traits. The size of males and females, as well as environmental potential of male-male competition, have been well recognized as modulators of sperm allocation in crustaceans in general [22,23] and crabs in particular [8,18,24,25]. Other factors, like mating history, also influence the amount of sperm delivered by males [5]. In wild populations, all these factors interact in the reproductive relations among crabs; therefore, a high variability could be expected in vasa deferentia condition from field individuals, where mating history and socio-sexual context is unknown. Despite these multiple sources of variability, in this study, localities with different fishing intensities displayed contrasting results, evidencing a significant effect of male-selective fishing on the seminal reserves and reproductive condition of males.

While the weight of the vasa deferentia represents the potential seminal material transferable to females, the VSI seems be a good indicator of the reproductive condition of males, because it is not highly affected by male size. Thus, it could be used in temporal, as well as spatial, intra-specific comparisons. For example, the VSI could be equivalent to the gonadosomatic index in females, indicating the time of year when mating occurs, its extension and intensity. This could be especially useful for deep sea crab species (like golden crabs) where direct visualization of mating is difficult and costly. However, effective comparison of VSI should be expressed in dry weight and estimates free of leg and chelae (as proposed by [6]) to increase accuracy.

At a temporal scale, seasonal variation in VSI was evident in Los Molinos, with lowest values during early spring and summer (September, December and January), but quickly recovering in late summer (February) to reach a maximum by fall (May). This finding is consistent with our records of mate guarding behavior in the field, which is almost exclusively found during spring and summer (mating period). On the other hand, females carry embryos during late fall and winter [15], therefore, males do not find receptive females in this period, which would allow recovery and/or maintenance of sperm reserves. It is necessary to note that changes in VSI also depend on changes in body mass and therefore, directly on the molt cycle of males. This cycle is poorly understood for this species, but should be studied further to make more accurate assessments of the reproductive process.

At a spatial scale, VSI obtained from *Metacarcinus edwardsii* showed a clear effect of fishing on reproductive condition of males. When the VSI obtained from males after experimental mating is compared with field VSI data, from early spring (September) until the end of summer (February), the poor reproductive potential of males from high fishing localities is put in evidence. Males from high fishery localities have a slight recovery in VSI, which did not reach the base line VSI of just-mated males from the lab. In contrast, males from low fishery localities showed higher VSI values than males following experimental matings. The large differences in the VSI of males from high and low localities was maintained regardless of the season, which indicate that this pattern is not related to well recognized latitudinal lag in reproductive periods [2].

An alternative explanation of the differences in VSI is that these findings reflect population abundance rather than effects of fishery on sex ratio of mature crabs. However, direct estimation of abundance using transect showed no association between VSI and crab abundances, low VSI were found in localities with very low (Ancud) and high (Dalcahue) crab abundances and higher VSI were also found in localities with contrasting population abundances (Los Molinos versus Calbuco).

Whether the poor reproductive potential of males from high fishing localities is able to cause sperm limitation in females is still an open question. The operational sex ratio is normally biased toward males, given that female receptivity is time-limited (i.e. a short period after molt) and males seem always ready to mate. Also, females may partly compensate parsimonious sperm allocation by individual males through polyandry, as evidenced by laboratory and field work with *Chionoecetes opilio* [26]. On the other hand, sperm limitation can be expected because: 1) due male selective fishing, the sex ratio in a population is severely skewed toward females. In this scenario, where males increase mating frequency, the outcome may be rapid sperm depletion, if sperm allocation is not tailored to mating opportunities, delivering less sperm to each mate [5,9,27]. *M*. *edwardsii* in a first mating event, transfers a mean of 15% of the seminal material stored in the vasa deferentia, very little in comparison to other species, like the blue crab *C*. *sapidus* (70% for large males, [21]), and subsequent mates would receive even less (LM Pardo unpublished data). The strategy of sperm allocation in this species seems be very conservative and vulnerable to sperm limitation, especially taking into account the high requirement of oocyte fertilization (800.000 eggs per brood from a female of 120 mm of carapace width, L.M. Pardo unpublished data). 2) Alternatively, if males strategically reduce sperm allocation to individual females, in order to inseminate as many females as possible (as demonstrated in [8]), then all females may be sub-optimally inseminated. 3) A large reduction in large males, which should dominate mating events, reduces the intensity of male competition for receptive females, allowing smaller males to mate. Small males may not deliver enough sperm to ensure oocyte fertilization, especially if these males had mated repeatedly [6,8]. Lastly, 4) pre-copulation guarding behavior, which is extensive in this species (up to 3 weeks), requires males to be larger than females, therefore, larger (and most fecund) females in this scenario would not have a suitable partner.

In this research, evidence of sperm depletion in a male-biased fishery is shown. Sperm depletion has been detected in harvested populations of other decapod species, namely the brachyuran blue crab (*Callinectes sapidus*) [21,28], in snow crab *(Chionoecetes opilio*) [8], the anomuran king crab (*Paralithodes brevipes*) [9,25] and the hermit crab (*Birgus latro*)[29]. All of these species are managed under the 3-S strategy (Season, Sex and Size control) fishing strategy. The rationale for this management is that harvesting above a minimum legal size provides at least one opportunity for males to mate with females. Harvest restrictions on females can protect partially or totally reproductively mature females and specific fishing seasons avoid harvesting crabs during mating and molting (soft-shell) periods. However, even with these precautionary measures, the 3-S management seems to affect the reproductive potential in males. In the case of the Chilean crab fishery, a ban seasonal could be applied during the mating season to provide some protection to males. Alternatively, non sex-selective harvesting strategy could be applied as in some other crab fisheries [30,31], and could be complemented with a maximum catch quota.
